# Optimizing the treatment mode for de novo metastatic nasopharyngeal carcinoma with bone-only metastasis

**DOI:** 10.1186/s12885-021-09152-1

**Published:** 2022-01-04

**Authors:** Cheng Lin, Sheng Lin, Lili Zhu, Shaojun Lin, Jianji Pan, Yun Xu

**Affiliations:** 1 Department of Radiation Oncology, Fujian Medical University Cancer Hospital, Fujian Cancer Hospital, No. 420 Fuma Road, Fuzhou, 350014 China; 2 Department of Medical Oncology, Fuqing City Hospital of Fujian, Fuqing, Fuzhou, 350300 China; 3 Department of Radiation Oncology, Fuzhou Pulmonary Hospital of Fujian, Fuzhou, 350008 China

## Abstract

**Background:**

No standard radiotherapy regimens have been established for the treatment of de novo metastatic nasopharyngeal carcinoma (mNPC) with bone-only metastasis. The current study aimed to investigate the efficacy of palliative chemotherapy (PCT) plus locoregional radiotherapy (LRRT) with or without local radiotherapy (RT) for metastatic bone lesions in mNPC.

**Methods:**

We retrospectively analysed 131 de novo patients with mNPC who had bone-only metastasis and received at least two cycles of PCT with LRRT. The difference in survival was evaluated by the log-rank test. Univariable and multivariable analyses were performed by Cox regression.

**Results:**

The median overall survival (OS) and progression-free survival (PFS) were 33.0 months and 24.0 months, respectively. Patients with five or fewer metastatic bone lesions had significantly longer OS (72.0 months vs. 23.0 months, Hazard ratios (HR) = 0.45, *p* <  0.001) and PFS (48.0 months vs. 15.0 months, HR = 0.52, *p* = 0.004) than those who had more than five metastatic bone lesions. Patients who received four or more cycles of chemotherapy were associated with significantly longer OS (unreached vs. 19.0 months, HR = 0.27, *p* <  0.001) and PFS (66 months vs. 16.0 months, HR = 0.32, *p* <  0.001). Multivariate analysis confirmed that fewer bone metastases (≤ 5) and more chemotherapy cycles (≥ 4) were favourable prognostic factors for OS. Subgroup analysis revealed that RT to metastatic bone lesions tended to prolong OS (83.0 months vs. 45.0 months) and PFS (60 months vs. 36.5 months) in patients with five or fewer metastatic bone lesions than in those without RT to metastatic bone lesions (*p* > 0.05). Patients who received a RT dose > 30 Gy had neither better OS (63.5 months vs. 32.0 months, *p* = 0.299) nor PFS (48.0 months vs. 28.0 months, *p* = 0.615) than those who received a RT dose ≤30 Gy.

**Conclusions:**

Local RT to bone metastases may not significantly improve survival in patients with de novo mNPC with bone-only metastasis who have already received PCT plus LRRT. Receiving four or more cycles of chemotherapy can significantly prolong survival and is a favourable independent protective factor.

**Supplementary Information:**

The online version contains supplementary material available at 10.1186/s12885-021-09152-1.

## Introduction

Nasopharyngeal carcinoma (NPC), with an incidence of up to 30 cases per 100,000 person-years [1], and approximately 4 to 10% of those patients have metastatic NPC (mNPC) at diagnosis [2]. mNPC is a heterogeneous entity that ranges from a single metastasis to multiple organ metastases. Bone metastasis is the most common type of organ metastasis, accounting for over 60% of all metastatic sites and favouring longer survival [3]. To date, mNPC is generally considered an incurable disease, and there is no optimal treatment. Palliative chemotherapy (PCT) is the primary treatment, and locoregional radiation therapy (LRRT) is strongly recommended in chemotherapy-sensitive patients with mNPC [4, 5]. Radiotherapy (RT) to metastatic bones is only widely administered for relieving pain and improving quality of life in patients with de novo mNPC who have bone-only metastasis.

Currently, an increasing number of studies have reported that patients with NPC who have solitary bone metastasis, or even with recurrent bone-only oligometastasis, could have long-term disease control and better survival [6, 7]. Moreover, emerging evidence has suggested a vital role for local RT in de novo mNPC with bone-only metastasis, giving fascinating insight into the management of mNPC with bone metastasis [8–11]. However, the potential benefit of combining PCT plus LRRT with or without RT to metastatic bone lesions in mNPC remains controversial. No consensus has been reached, and no standard regimens have been strongly recommended [2, 4].

In the present study, we retrospectively analysed 131 patients with de novo mNPC who had bone-only metastasis between June 2007 and December 2017 at our cancer centre and explored the clinical significance of different practice strategies in de novo mNPC with different patterns of bone metastasis (metastatic bone sites ≤5 and > 5), which was aimed to optimize the treatment regimens and find the most potential candidates.

## Materials and methods

### Patients

A total of 131 patients with mNPC were admitted to Fujian Cancer Hospital between June 2007 and December 2017. The inclusion criteria were as follows: (I) patients with newly and histologically diagnosed mNPC; (II) mNPC with bone-only metastasis; (III) patients had two or more cycles of chemotherapy; and (IV) Eastern Cooperative Oncology Group (ECOG) performance score ≥ 1. The exclusion criteria were as follows: (I) patients with NPC who developed multiple organ metastases; (II) patients who were previously treated; (III) patients who were lost to follow-up; or (IV) patients who had less than two cycles of chemotherapy. Regarding the diagnostic procedure and criteria of bone metastases, patients were first screened by emission computed tomography (ECT) of bones. Then, the result was further confirmed by at least one of the following examinations: computed tomography (CT) with contrast, magnetic resonance imaging (MRI) with contrast, positron emission tomography-computed tomography (PET/CT) or pathological diagnosis. Restaging of all patients was performed according to the 8th edition of the American Joint Committee on Cancer (AJCC)/Union for International Cancer Control (UICC). Our study was approved by the Ethics Committee of Fujian Medical University Cancer Hospital, Fuzhou, China. Written informed consent was obtained from all patients.

### Treatment

All patients received platinum-based systematic chemotherapy. Chemotherapy regimens, including gemcitabine, paclitaxel, or docetaxel plus platinum, were administered every 3–4 weeks. LRRT to the nasopharynx and neck was conducted by two-dimensional radiotherapy (2D-RT) or intensity modulated radiotherapy (IMRT), which was described previously [12]. A total of 38.2% (50/131) of patients received RT to metastatic bones. 2D-RT, IMRT, volumetric modulated arc therapy (VMAT), or tomotherapy were used for RT of bone metastases. Of the 50 patients who received RT for bone metastases, 90% (45/50) of them received radiation to all their bone metastatic lesions. The others received partial radiation to relieve bone pain. The patterns of RT to metastatic bone lesions were heterogeneous; 60% (30/50) of patients received 30 Gy with 10 fractions, and 30% (14/50) of patients received 40 Gy with 20 fractions. Six patients received 45–70 Gy irradiation (2 Gy/fraction) for nonspinal bone metastases. Treatment-related grade 3 or 4 adverse events occurred in 38.9% (51/131) of all patients. No treatment-related deaths occurred.

### Follow-up

Evaluation of tumour response, including CT, MRI, ECT or PET/CT, was selectively conducted after every two or three cycles of chemotherapy. After all therapeutic processes, patients were evaluated every 3 months for the first 2 years, every 6 months from years 3–5, and then every 12 months. Overall survival (OS) was measured from the date of diagnosis to the date of death from any cause. Progression-free survival (PFS) was measured from the date of diagnosis to the time of disease progression or death from any cause.

### Statistical analysis

All statistical analyses were performed using the software SPSS version 24.0 and Graph Pad Prism 8. The Cox regression model was used for the univariate analysis and multivariate analysis. The median follow-up time was calculated by reverse Kaplan-Meier analysis. Kaplan–Meier analysis and log-rank method were used to compare survival differences. *p* values < 0.05 were considered statistically significant, and all p values were two-sided.

## Results

### Patient characteristics

**Fig. 1:**
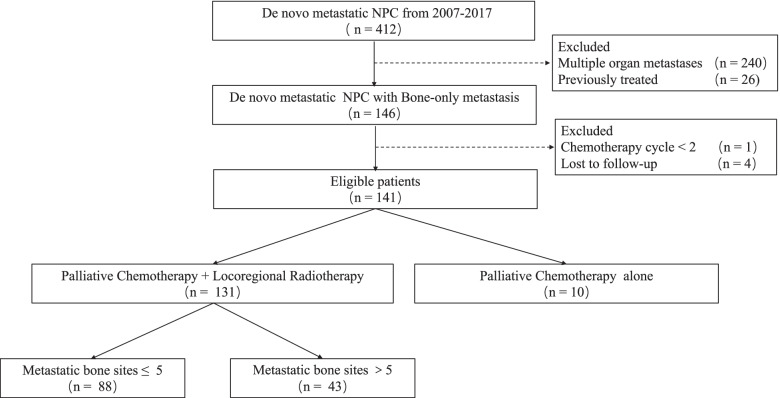
Flow diagram of study selection process

A total of 131 patients with de novo mNPC who had bone-only metastasis who were treated with PCT plus LRRT and RT to metastatic bone lesions between January 2007 and December 2017 were eligible for our study (Fig. 1).

**Fig. 2:**
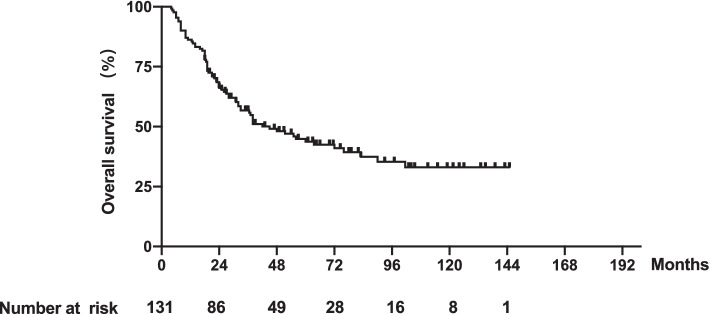
Kaplan-Meier curves of OS in 131 patients with de novo metastatic NPC with bone-only metastasis

The median age was 46.4 years (range 17–73 years). The median follow-up time was 71.5 months (95% confidence interval (CI), 57.6–85.4 months). The median OS was 33.0 months (range 4–145 months); the median PFS was 24.0 months (range 2–145 months); and the 1-, 3- and 5-year survival rates were 85.5, 55.8 and 43.7%, respectively (Fig. 2).

The baseline characteristics of the 131 patients who were eligible are shown in Table 1.

**Table 1:** Characteristics of 131 de novo mNPC with Bone-only metastasis

Characteristics	RT to bone metastases		***p***
	No n (%)	Yes n (%)	
Total	81 (61.8)	50 (38.2)	
Age(y)			0.270
≤ 50	49 (60.5)	35 (70.0)	
> 50	32 (39.5)	15 (30.0)	
Sex			0.941
Female	66 (81.5)	41 (82.0)	
Male	15 (18.5)	9 (18.0)	
T stage			0.458
T1–2	26 (32.1)	13 (26.0)	
T3–4	55 (67.9)	37 (74.0)	
N stage			0.907
N0–1	13 (16.0)	9 (18.0)	
N2–3	68 (84.0)	41 (82.0)	
ECOG score			1.000
0	72 (88.9)	46 (92.0)	
1	9 (11.1)	4 (8.0)	
Chemo cycles			0.417
< 4	26 (32.1)	16 (32.0)	
≥ 4	55 (67.9)	34 (68.0)	
No. of bone metastasis			0.480
≤ 5	50 (61.7)	38 (76.0)	
> 5	31 (38.3)	12 (24.0)	
IMRT^a^			0.419
No	34 (42.0)	19 (38.0)	
Yes	47 (58.0)	31 (62.0)	

*ECOG* Eastern Cooperative Oncology Group, *Chemo* Chemotherapy, *RT* radiotherapy, ^a^RT techniques for nasopharynx and neck

In all, there were 67.2% (88/131) of patients who had five or fewer metastatic bone lesions; 38.2% (50/131) of patients received RT to bone metastases; and there were 67.9% (89/131) of patients who had received 4 or more cycles of chemotherapy.

### Comparison of survival in mNPC with different bone metastatic lesions

**Fig. 3:**
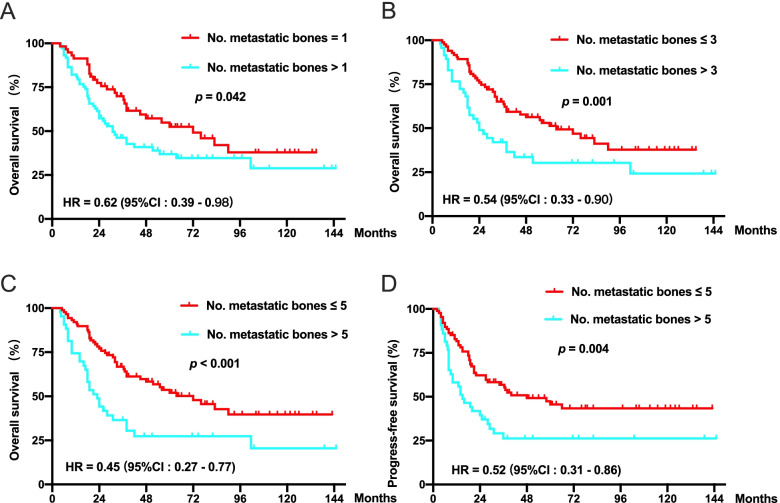
Kaplan-Meier curves for OS or PFS in 131 patients with de novo metastatic NPC classified by different cutoff values of metastatic bone lesions. **a** Patients grouped according to 1 metastatic bone lesion. **b** Patients grouped according to 3 metastatic bone lesions. **c** and **d** Patients grouped according to 5 metastatic bone lesions

To explore whether LRRT can benefit patients who receive PCT plus LRRT, a subgroup analysis was performed according to the number of bone metastases. Cut-off values of 1, 3 and 5 were all significant in predicting OS (Fig. 3a-c). Compared with the cut-off values of 1 and 3, the cut-off value of 5 had the minimum HR value. Therefore, 5 was defined as the cut-off value of the number of bone metastases. Patients with five or fewer metastatic bone lesions had significant associations with prolonged OS (72.0 months vs. 23.0 months, HR = 0.45, *p* <  0.001) and PFS (48.0 months vs. 15.0 months, HR = 0.52, *p* = 0.004) (Fig. 3c and d).

Univariate and multivariate analyses further confirmed that fewer metastatic bone lesions (≤ 5) were a favourable prognostic factor for OS (Table 2). In addition, receiving 4 or more chemotherapy cycles predicted better survival outcomes.

**Table 2:** Univariable and Multivariate analysis for PFS and OS in 131 de novo mNPC patients

	Univariable		Multivariable	
	HR (95% CI)	*p*	HR (95% CI)	*p*
**Progress-free survival**				
Age (≤ 50 vs > 50)	1.184 (0.742–1.889)	0.479		
Sex (Female vs Male)	0.925 (0517–1.655)	0.792		
Chemotherapy cycles (< 4 vs ≥ 4)	0298 (0.186–0.477)	< 0.001	0.370 (0.215–0.638)	< 0.001
IMRT (No vs Yes)	0.480 (0.303–0.759)	0.002	0.785 (0.461–1.336)	0.371
No. of bone metastasis (≤ 5 vs > 5)	0.503 (0.316–0.800)	0.004	0.597 (0.383–0.930)	0.053
RT to bone metastases (No vs Yes)	0.988 (0.620–1.577)	0.961		
**Overall Survival**				
Age (≤ 50 vs > 50)	1.600 (1.009–2.537)	0.046	1.484 (0.929–2.372)	0.099
Sex (Female vs Male)	1.010 (0.572–1.781)	0.973		
Chemotherapy cycles (< 4 vs ≥ 4)	0.269 (0.170–0.425)	< 0.001	0.379 (0.224–0.639)	< 0.001
IMRT (No vs Yes)	0.374 (0.235–0.595)	< 0.001	0.624 (0.369–1.054)	0.078
No. of bone metastasis (≤ 5 vs > 5)	0.449 (0.282–0.715)	0.001	0.567 (0.352–0.915)	0.020
RT to bone metastases (No vs Yes)	0.914 (0.572–1.459)	0.706		

### Efficacy of additional RT to metastatic bone lesions in patients who received PCT plus LRRT

**Fig. 4:**
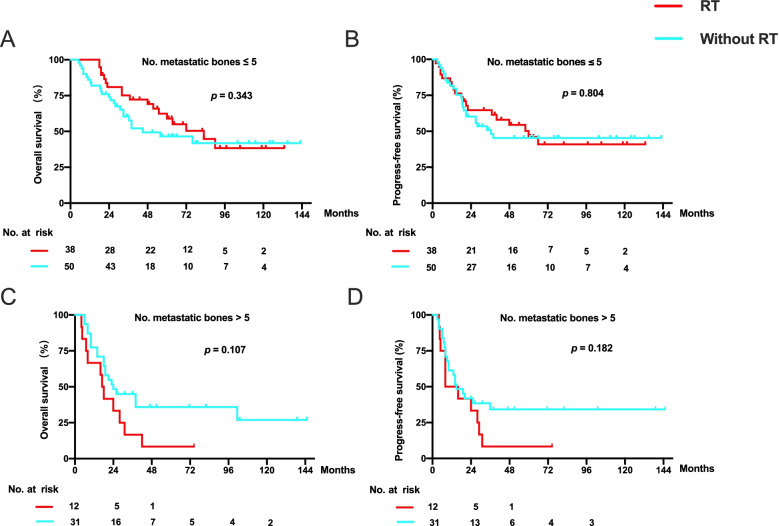
Kaplan-Meier curves for OS and PFS of 88 patients with de novo metastatic NPC with five or fewer metastatic bone lesions (**a** and **b**) and 43 patients with de novo metastatic NPC with more than five metastatic bone lesions (**c** and **d**) based on whether patients received RT to metastatic bone lesions

To address whether RT to metastatic bone lesions would generate actual benefits in patients with de novo mNPC who had different numbers of metastatic bone lesions, patients were stratified by the number of metastatic bone sites (≤ 5 vs. > 5). The data revealed that despite the trend of benefit, RT to metastatic bone sites had no statistical significance in OS (83.0 months vs. 45.0 months, *p* = 0.343) and PFS (60 months vs. 36.5 months, *p* = 0.804) in patients with five or fewer metastatic bone lesions (*p* > 0.05) compared to those without RT to metastatic bone lesions (Fig. 4a and b). Application of RT to metastatic bone lesions also failed to bring any survival benefit in patients with less than five metastatic bone lesions (Fig. 4c and d).

Similar results were found according to the cut-off values of 1 (Supplementary Fig. S1a and b) and 3 (Supplementary Fig. S1c and d) bone metastases. Univariable and multivariate analysis for OS classified by the number of metastatic bone sites (≤ 5 vs. > 5) revealed that RT to metastatic bone lesions was not an independent predictive factor (Supplementary Table S1).

### Efficacy of RT dose prescription and chemotherapy cycles in patients who received PCT plus LRRT

**Fig. 5:**
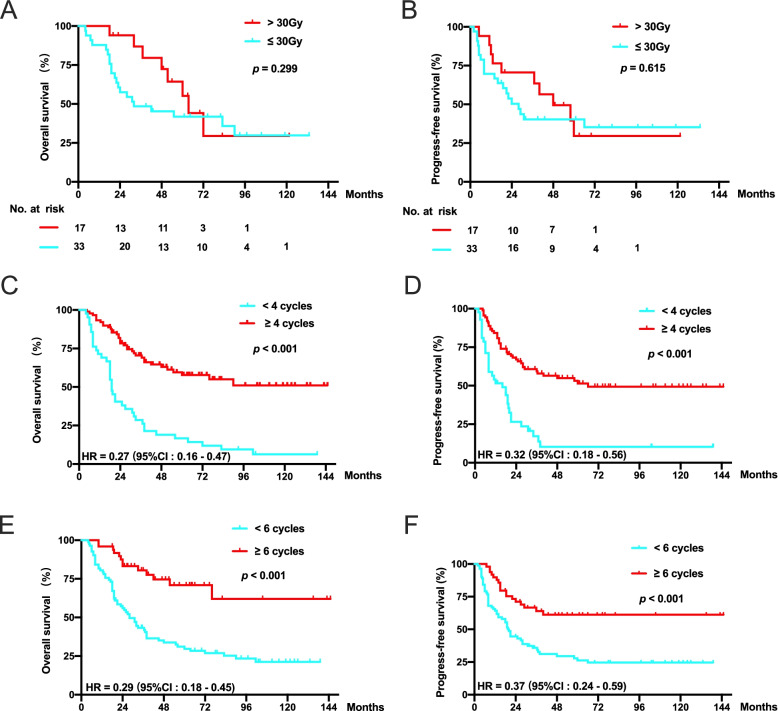
Kaplan-Meier curves for OS (**a**) and PFS (**b**) according to a radiotherapy dose > 30 Gy and a radiotherapy dose ≤30 Gy in 50 patients with de novo mNPC receiving radiotherapy to metastatic bone lesions. Kaplan-Meier curves for OS and PFS according to 4 cycles (**c** and **d**) and 6 cycles (**e** and **f**) of chemotherapy

To evaluate whether RT dose affects the outcomes, RT dose prescriptions were classified into two groups (≤ 30 Gy and > 30 Gy). Compared to patients who received more than a RT dose prescription > 30 Gy, those who received a RT dose prescription of ≤30 Gy tended to have a worse OS (63.5 months vs. 32.0 months) and PFS (48.0 months vs. 28.0 months). Of note, no significant difference was found (Fig. 5a and b).

As systemic therapy was the fundamental treatment in mNPC, we further explored the impact of chemotherapy cycles in patients with bone-only mNPC. In our study, 37.4% (49/131) and 67.9% (89/131) of patients received ≥6 cycles or ≥ 4 cycles of chemotherapy, respectively. Considering the safety and tolerability for patients receiving chemotherapy and the cut-off value of 4 having the minimum HR value, chemocycles were separated by 4 cycles instead of 6 cycles. We found that patients receiving 4 or more cycles of chemotherapy had a significant association with longer OS (unreached vs. 19.0 months) and PFS (66 months vs. 16.0 months) (Fig. 5c and d). In addition, patients who received ≥6 cycles of chemotherapy also had longer OS (unreached vs. 28.0 months) and PFS (unreached vs. 20.0 months) than those who received < 6 cycles of chemotherapy (Fig. 5e and f).

## Discussion

Treatment of mNPC is a major challenge for RT physicians. The skeleton is the most common site of distant metastasis in NPC, whereas the optimal therapeutic strategy has remained largely undefined. Our study showed that patients with mNPC who had five or fewer metastatic bone lesions had improved OS and PFS. There was no significant benefit from palliative RT to metastatic bone lesions in patients with mNPC who had bone-only metastasis. Patients benefited most from receiving 4 or more cycles of chemotherapy. Fewer metastatic bones (≤ 5) and more cycles of chemotherapy (≥ 4) were proven to be independent favourable protective factors.

Local RT for mNPC is becoming a hot-button issue [13–15]. A phase 3 randomized clinical trial demonstrated that palliative chemotherapy (PCT) plus locoregional radiotherapy (LRRT) can significantly prolong OS in chemotherapy-sensitive patients with mNPC [5]. In addition, a new study reported that local treatment of metastases could improve the OS of patients with mNPC, regardless of metastatic sites and the number of metastatic lesions [16]. However, for patients with mNPC with bone-only metastasis who had already received PCT combined with LRRT, whether additional RT to metastatic bone lesions will bring survival benefit has not yet been well characterized. Li et al. reported that patients with mNPC who received intensive local RT to bone lesions had longer OS (HR = 0.63) and PFS (HR = 0.80) [9]. The article also suggested that RT with palliative dose prescription to metastatic bone lesions was not recommended in patients with bone metastatic NPC. Consistent with Li’s report, our study indicated that patients receiving RT with a palliative dose prescription who had five or fewer metastatic bone lesions had no significant association with longer OS or PFS, although a tendency of survival benefit was seen. In addition, our study suggested that patients who received a RT dose prescription > 30 Gy tended to have better survival than those who received a RT dose prescription ≤30 Gy. Of note, Li’s study included patients with metastases to organs other than bone, while our study only included patients with mNPC with bone-only metastasis. Taken together, the role of additional RT in metastatic bone disease is of great interest and warrants further research.

For patients with mNPC, adequate systemic chemotherapy is strongly recommended as the first-line treatment. Concerning the optimum cycles of chemotherapy in mNPC, several retrospective studies have suggested that patients receiving ≥4 cycles of systemic chemotherapy had longer OS than those who received 1–3 cycles of systemic chemotherapy [9, 17–20]. Chen et al. reported that, compared to < 6 cycles of chemotherapy, no significant survival benefit was observed from ≥6 cycles of chemotherapy in de novo mNPC [21]. In contrast, significantly longer survival was achieved by patients with mNPC with synchronous liver metastasis having ≥6 cycles of chemotherapy compared to those receiving < 6 cycles of chemotherapy [22]. In line with most studies, our study indicated that patients benefit the most from receiving ≥4 cycles of systemic chemotherapy (HR = 0.23). It is worth noting that we also found that patients who received ≥6 cycles of chemotherapy also benefited in OS and PFS compared with those who received < 6 cycles of chemotherapy. In summary, for patients with mNPC with bone-only metastasis, ≥ 4 cycles of systemic chemotherapy may be considered if patients can tolerate the side effects of chemotherapy and RT.

The study had some limitations. First, our study was a retrospective study in a single centre. Second, our sample sizes were relatively small, which might affect statistical performance. Third, only 4 patients received a RT dose prescription > 60 Gy, which could affect our evaluation of the role of RT in metastatic bone lesions. EBV DNA and other blood biomarkers were not assessed in our study. Further prospective trials are needed in the future to guide the management of de novo mNPC with bone-only metastasis.

## Conclusions

For patients with mNPC with bone-only metastasis who have already received PCT combined with LRRT, RT to metastatic bone lesions may not significantly improve survival. Receiving four or more cycles of chemotherapy is strongly recommended. Prospective clinical trials are expected to confirm these results and to find the optimal population.

## Supplementary Information

**Additional file 1: Figure S1** Kaplan-Meier curves for OS of 131 patients with de novo metastatic NPC based on whether patients received RT to metastatic bone lesions or not that was divided by the cut-off values of 1 (a and b) and 3 bone metastases (c and d).

**Additional file 2: Supplementary Table 1** Univariable and Multivariate analysis for OS in de novo mNPC patients classified by number of metastatic.

## Data Availability

The datasets generated and/or analyzed in our study are available from the corresponding author.

## Abbreviations

RT: Radiation therapy
LRRT: Locoregional radiation therapy
mNPC: Metastatic nasopharyngeal carcinoma
PCT: Palliative chemotherapy
IMRT: Intensity-modulated radiotherapy
OS: Overall survival
PFS: Progression-free survival
PSM: Propensity score-matched
ECOG: Eastern Cooperative Oncology Group
AJCC: American Joint Committee on Cancer
UICC: Union for International Cancer Control
HR: Hazard ratios
CI: Confidence interval
EBV: Epstein-Barr virus
MRI: Magnetic resonance imaging
ECT: Emission computed tomography
PET/CT: Positron emission tomography-computed tomography
