# Supplementary material for: Optimizing the treatment mode for de novo metastatic nasopharyngeal carcinoma with bone-only metastasis
Source: BMC Cancer. 2022 Jan 4;22:35. doi: 10.1186/s12885-021-09152-1 (PMC8729074; doi:10.1186/s12885-021-09152-1)
Supplement: Supplementary file 1 — Additional file 1: Figure S1 Kaplan-Meier curves for OS of 131 patients with de novo metastatic NPC based on whether patients received RT to metastatic bone lesions or not that was divided by the cut-off values of 1 (a and b) and 3 bone metastases (c and d). [file 12885_2021_9152_MOESM1_ESM.pdf]

Supplementary Figure S1

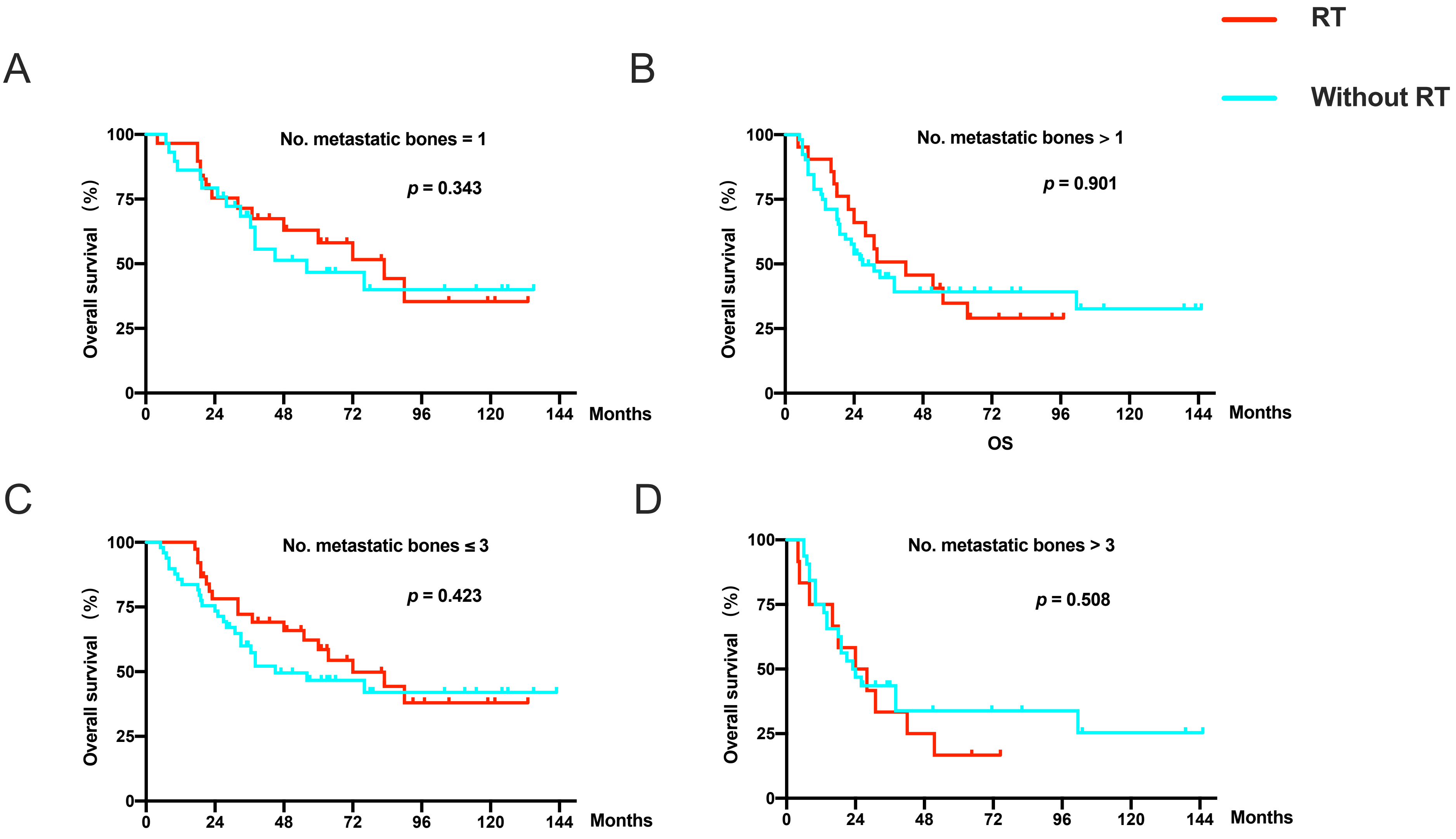

Fig. S1 Kaplan-Meier curves for OS of 131 patients with de novo metastatic NPC based on whether patients received RT to metastatic bone lesions or not that was divided by the cut-off values of 1 (a and b) and 3 bone metastases (c and d).
