# Supplementary material for: Optimizing the treatment mode for de novo metastatic nasopharyngeal carcinoma with bone-only metastasis
Source: BMC Cancer. 2022 Jan 4;22:35. doi: 10.1186/s12885-021-09152-1 (PMC8729074; doi:10.1186/s12885-021-09152-1)
Supplement: Supplementary file 2 — Additional file 2: Supplementary Table 1 Univariable and Multivariate analysis for OS in de novo mNPC patients classified by number of metastatic. [file 12885_2021_9152_MOESM2_ESM.doc]

**Supplementary table 1** Univariable and Multivariate analysis for OS in de novo mNPC patients classified by number of metastatic bone sites (≤ 5 vs. > 5)

|  | **Univariable** | | |  | **Multivariable** | |
| --- | --- | --- | --- | --- | --- | --- |
|  | HR (95% CI) | *p* | |  | HR (95% CI) | *p* |
| **No. metastatic bones ≤ 5** |  | |  |  |  |  |
| Age (≤ 50 vs > 50) | 1.770 (0.969-3.235) | | 0.063 |  |  |  |
| Sex (Female vs Male) | 0.987 (0.473-2.061） | | 0.973 |  |  |  |
| Chemotherapy cycles (<4 vs ≥ 4) | 0.338 (0.185-0.617) | | < 0.001 |  | 0.379 (0.224-0.639) | < 0.001 |
| IMRT (No vs Yes) | 0.374 (0.235-0.595) | | < 0.001 |  | 0.624 (0.369-1.054) | 0.078 |
| RT to bone metastases (No vs Yes) | 0.747 (0.407-1.371) | | 0.346 |  |  |  |
| **No. metastatic bones > 5** |  | |  |  |  |  |
| Age (≤ 50 vs > 50) | 1.770 (0.969-3.235) | | 0.063 |  |  |  |
| Sex (Female vs Male) | 1.177 (0.481-2.879） | | 0.721 |  |  |  |
| Chemotherapy cycles (<4 vs ≥ 4) | 0.490 (0.240-1.002) | | 0.051 |  |  |  |
| IMRT (No vs Yes) | 0.507 (0.244-1.503) | | 0.069 |  |  |  |
| RT to bone metastases (No vs Yes) | 0.747 (0.407-1.371) | | 0.077 |  |  |  |
